# Negative Influence by the Force: Mechanically Induced Hyperpolarization via K_2P_ Background Potassium Channels

**DOI:** 10.3390/ijms22169062

**Published:** 2021-08-23

**Authors:** Miklós Lengyel, Péter Enyedi, Gábor Czirják

**Affiliations:** Department of Physiology, Semmelweis University, P.O. Box 2, H-1428 Budapest, Hungary; lengyel.miklos@med.semmelweis-univ.hu (M.L.); enyedi.peter@med.semmelweis-univ.hu (P.E.)

**Keywords:** mechanosensitive, potassium channel, membrane tension, stretch, KCNK2, KCNK4, KCNK10, TREK1, TREK2

## Abstract

The two-pore domain K_2P_ subunits form background (leak) potassium channels, which are characterized by constitutive, although not necessarily constant activity, at all membrane potential values. Among the fifteen pore-forming K_2P_ subunits encoded by the KCNK genes, the three members of the TREK subfamily, TREK-1, TREK-2, and TRAAK are mechanosensitive ion channels. Mechanically induced opening of these channels generally results in outward K^+^ current under physiological conditions, with consequent hyperpolarization and inhibition of membrane potential-dependent cellular functions. In the past decade, great advances have been made in the investigation of the molecular determinants of mechanosensation, and members of the TREK subfamily have emerged among the best-understood examples of mammalian ion channels directly influenced by the tension of the phospholipid bilayer. In parallel, the crucial contribution of mechano-gated TREK channels to the regulation of membrane potential in several cell types has been reported. In this review, we summarize the general principles underlying the mechanical activation of K_2P_ channels, and focus on the physiological roles of mechanically induced hyperpolarization.

## 1. Introduction

**Figure 1:**
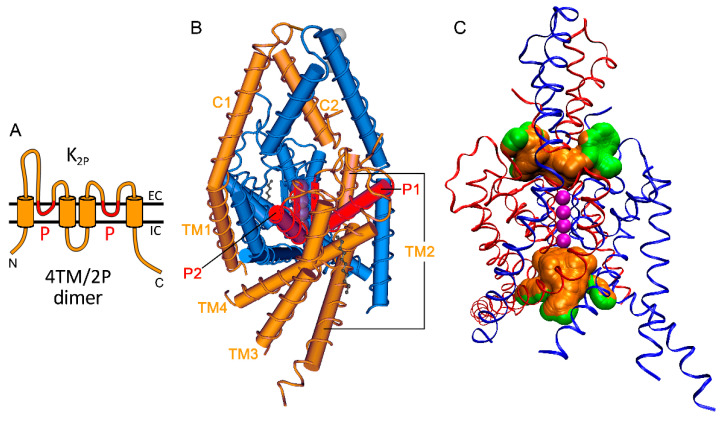
Schematic overview of the structure of K_2P_ background potassium channels. (**A**) The general transmembrane topology of K_2P_ channels is 4TM/2P. Only one subunit of the dimer is shown. The membrane is illustrated with two horizontal black lines, both the N- and C-terminus of the subunit (N and C) are intracellular (IC). The subunit contains two pore domains (P), which form reentrant loops on the extracellular (EC) side (indicated with red). A part of the long first extracellular loop, N-terminal to the first pore domain, constitutes the extracellular cap structure. (**B**) The panoramic view of the crystal structure of TREK-2 homodimer is shown from the extracellular side (PDB: 4BW5). One subunit is orange (pore loops are red) and the other is blue, and the α-helical regions are illustrated as tubes. The first transmembrane segment (TM1) is followed by the two cap helices (C1 and C2). After C2, the first pore helix (P1) diagonally immerses into the transmembrane space, and the chain vertically returns to the EC side as the (TVGYG-like) signature sequence of the selectivity filter (narrow rectangle close to the K^+^ binding sites, illustrated with spheres). The pore helix and the signature sequence belong to the first pore domain, as illustrated in panel A. The amino acid chain continues as the (not entirely straight) TM2 and TM3 transmembrane helices, followed by the second pore domain, which includes the P2 pore helix and the other signature sequence rotated by approx. 90 degrees around the axis of the pore, compared to the first one. Finally, the chain returns to the IC side as the TM4 transmembrane helix. Some coils connecting the helical elements (e.g., between C2 and P1, TM2 and TM3) and the long C-terminal tail following TM4 are missing from the crystal structure. The figure was produced by the Cn3D software of NCBI. (**C**) The extracellular ion pathway (EIP) below the cap structure connects the EC end of the transmembrane pore to the EC space as a T-shaped bifurcation. The narrow selectivity filter region of the transmembrane pore is illustrated as a series of four K^+^ binding sites (purple spheres). Below the selectivity filter, the pore widens as the central cavity. The TREK-2 homodimer is rotated by about a quarter turn, compared to *panel B*, the cap helices of the two subunits almost overlap in the illustration. The EIP and central cavity are determined as the spaces accessible by a small sphere (radius 2.1 Å), but not by a large one (radius 4.5 Å for the EIP and 8.1 Å for the central cavity), by using PrinCCes [10] and VMD [11] software. The green spheres are adjacent to bulk fluid.

The two-pore domain (K_2P_) potassium channels are the molecular correlates of background (leak) potassium currents, which mediate K^+^ transport through the plasma membrane, regulate the value of the membrane potential, and adjust cellular excitability [1]. The different K_2P_ channel types are characterized by similar membrane topology, molecular architecture, and overall electrophysiological properties. The K_2P_ channels have four transmembrane segments and two pore domains in a subunit (4TM/2P architecture, Figure 1A). As in the other families of potassium channels, the selectivity filter of K_2P_ channels is constituted by the TVGYG-like signature sequences of four pore domains, accordingly, assembled by the dimerization of two-pore domain subunits (Figure 1B), and considered as the major location of channel gating [2,3,4,5,6,7,8,9].

The first extracellular loop of K_2P_ channels is longer than the second one; this first loop contains conserved α-helices to establish a cap structure, through which the extracellular ion pathway (EIP) provides access to the narrow outer end of the transmembrane pore (Figure 1B,C) [12,13,14]. Close to the intracellular opening of the pore, below the selectivity filter, a wide central cavity is formed by the diverging transmembrane helices (Figure 1C). This cavity, just like the middle part of the EIP, may often accommodate a K^+^ ion partially retaining its hydration shell. The K^+^ ion in the central cavity may be stabilized by the electric dipole moment of the pore helices, whereas in the middle part of the EIP by negatively charged amino acid side chains in some K_2P_ channel types (such as TASK-3 or TREK-2) [12,13,15,16].

K_2P_ channels have background (K^+^-selective leak) characteristics, their macroscopic current is much better approximated by the Goldman–Hodgkin–Katz (GHK) current equation than the current of the voltage-gated (K_V_) or inwardly rectifying (K_IR_) channel types. In accordance with this equation, the current–voltage (I–V) relationship of the macroscopic current would be a line passing through the origin in symmetrical [K^+^], and under physiological conditions, the apparent outward rectification would be exclusively caused by the unequal distribution of K^+^ on the two sides of the membrane. Nevertheless, most K_2P_ channels deviate from this theoretical relationship to some extent (e.g., the members of the TREK subfamily show additional outward rectification of the macroscopic current). This is caused (at least in part) by the voltage-dependent gating of K_2P_ channels, which has recently been attributed to the ion flux-coupled gating mechanism [2].

Since the K_2P_ subunits do not contain a voltage–sensor transmembrane segment (as opposed to the K_V_ voltage-gated K^+^ channels), the charge carrier itself, the K^+^ ion in the channel pore has been suggested to be responsible for the voltage-dependence. The inward flux of K^+^ results in the emptying of the selectivity filter potassium binding sites, followed by the closure of the pore at this region, whereas the outward K^+^ flux replenishes the sites and favors the open state. This valve-like function explains the voltage-dependent gating with fast activation and deactivation kinetics in the low ms range, and may appear as the additional outward rectification at the level of the macroscopic current [2]. In the case of TREK-1 (but not TREK-2 or TRAAK), the outward rectification becomes even more prominent in the presence of extracellular Mg^2+^ or Ca^2+^, probably reflecting the blockage of the pore by these divalent ions from the extracellular side [17]. The members of the TREK subfamily are not constantly open, but gate with a rather low probability of the open state (p_o_) under resting conditions. As detailed above, p_o_ is increased by depolarization to some extent, furthermore it is also robustly regulated by other voltage-independent factors (e.g., by mechanical stimuli in the case of the TREK/TRAAK channels).

The K_2P_ channels are classified into six subfamilies (TWIK, TREK, TASK, TALK, THIK, and TRESK), based on sequence similarity and functional characteristics. The members of the different subfamilies share low (typically < 30%) amino acid sequence identity [18], and in general, the transmembrane segments are the most highly conserved regions. In good accordance with the practically missing similarity, apart from the transmembrane segments, the members of the different subfamilies are regulated by highly diverse physicochemical factors and intracellular signaling mechanisms. Therefore, the regulatory properties of the members of the TREK subfamily, detailed in this review, cannot be generalized to the other K_2P_ channels, so the other K_2P_ subfamilies have to be considered as completely different entities with respect to regulation. Within the subfamilies, the similarity is more pronounced. For example, in the TREK subfamily, the amino acid identity between TREK-1 (KCNK2 gene, K_2P_2.1 protein) and TREK-2 (KCNK10, K2P10.1) is 65%, and between TREK-2 and TRAAK (KCNK4, K2P4.1) is 45% [19]. Furthermore, the TREK/TRAAK channels also share several functional properties.

Whereas some mechanosensitive channel types (e.g., the Piezo1 nonselective cation channel) are primarily dedicated to mechanosensation [20,21], the members of the TREK subfamily are multimodal integrators of a wide variety of regulatory parameters, among which mechanosensitivity is only one factor. It is a general rule that the regulatory parameters interact, and the activation of a given regulatory pathway influences the sensitivity to the other stimuli [1]. Accordingly, the mechanosensitivity of TREK/TRAAK channels is substantially modified by several other regulatory mechanisms. The strong stimulation by another pathway may limit the mechanosensitivity in the sense that the already active channels cannot be further activated to a large extent. At lower non-mechanical stimulus intensities, the potentiation of mechanosensitivity and the shift of the stimulus–response curve toward less intense mechanical stimuli may appear (i.e., the channel is opened more easily by the mechanical effect [22]). 

The members of the TREK subfamily are characterized by the relatively short intracellular N-terminus, short loop region between the second and third transmembrane segments, and an extended (typically around 120–220 amino acid) intracellular tail region at the C-terminal part of the K_2P_ subunit (Figure 1A). The N-terminus is varied by both alternative splicing and translation initiation (ATI), and the latter determines the single channel conductance of TREK-1 and TREK-2 [23,24]. The long C-terminal tail region is missing from the crystal structures; nonetheless, it has paramount importance in the polymodal regulation of TREK/TRAAK channels. Several regulatory factors influence channel activity via the interaction with the C-terminal tail (Ct) [1].

A reported example is the activation of TREK-1 and TREK-2 channels by intracellular acidification, which has been shown to depend on the protonation of a specific glutamate residue (E306 in TREK-1) in the proximal C-terminal tail (pCt) [25,26]. According to a plausible model, the protonation of the glutamate residue in the pCt eliminates a negative charge, and facilitates the interaction between the pCt and the inner negative surface of the plasma membrane. This results in the relocation of the intracellular end of the fourth transmembrane segment (TM4) and induces channel opening. In contrast, the phosphorylation of the pCt by protein kinase A and C (S333 and S300 in TREK-1, respectively) inhibits the channel, possibly by impeding the interaction between the pCt and the plasma membrane [27,28,29].

Interestingly, the relationship of the pCt and channel gating appears to be different in TRAAK from TREK-1 and TREK-2. TRAAK is not activated by intracellular acidification; although it contains glutamate in the relevant position [25], it is not influenced by PKA- or PKC-dependent phosphorylation [30], and pCt appears to stabilize a less active conformation of TRAAK under resting conditions than in TREK-1 and TREK-2 [31].

The members of the TREK subfamily are activated by arachidonic acid and other polyunsaturated fatty acids (PUFA) [30,32,33], thermosensitive in the physiological range in intact cells (but not in excised patches) [34,35,36], and TREK-1 (but not TREK-2 and TRAAK) is inhibited by spadin, the secreted peptide belonging to the neurotensin receptor 3 (NTSR3/Sortilin) system [37,38,39]. TREK channels are also robustly regulated by protein interaction partners binding to the Ct. A-Kinase Anchoring Protein 150 (AKAP150), substantially increases channel activity, and prevents further activation by mechanical and other regulatory mechanisms [40]. The TREK channels are also targeted by pharmacological agents, volatile anesthetics increase the K^+^ current [29,41,42,43], and the antidepressant fluoxetine is an inhibitor of TREK-1 and TREK-2 [44,45,46,47,48,49]. The above wide range of factors are not subtle modulators, but one by one evokes a robust effect on TREK/TRAAK channel activity. Therefore, in the case of the TREK/TRAAK channels, the mechanosensitivity should always be considered by taking into account the other regulatory parameters. While TREK/TRAAK mechanosensitivity is clearly important in some tissues (see below), elsewhere, the mechanical stimulation appears to be less probable and the channels are mainly controlled by other regulatory factors. The members of the TREK subfamily are abundantly expressed at several locations (e.g., widespread expression in the neurons of the different brain regions [50,51,52,53,54], or high TREK-1 expression in the human adrenal gland [55,56]), where it is far from certain whether, or how, the cellular responsiveness to mechanical stimuli contributes to the physiological function.

## 2. Mechanical Activation of K_2P_ Channels

### 2.1. A General Approach to Mechanosensitivity of K_2P_ Ion Channels

**Figure 2:**
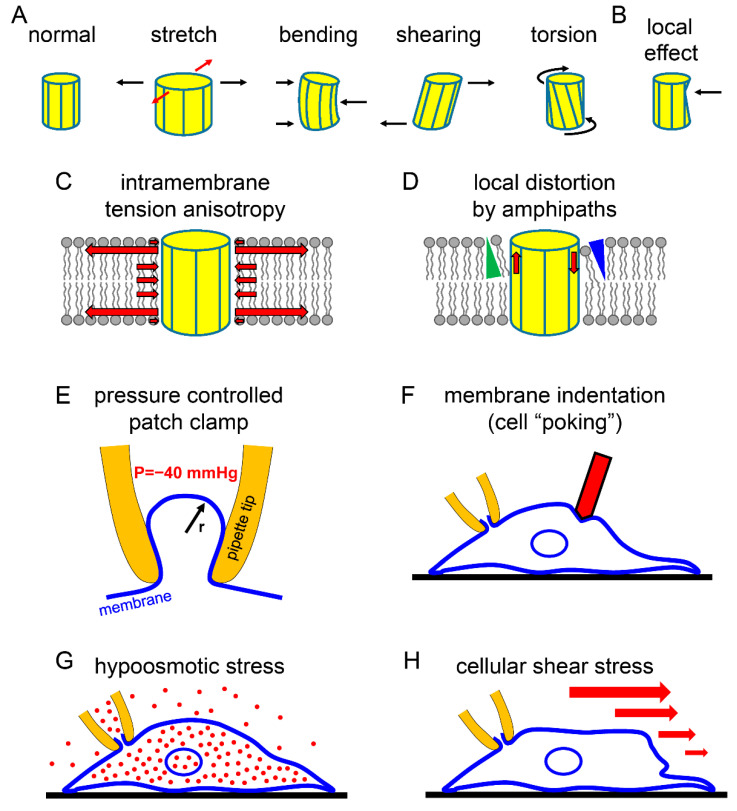
Global and local mechanical effects may influence the ion channel proteins, and the generally used methods to study mechanosensitive ion channels induce membrane tension (stretch) with a not particularly well-defined combination of the other effects. (**A**) Possible global mechanical effects have the potential to influence the whole channel protein complex. (**B**) The force may be transmitted to the channel protein through a specific molecular determinant as a local effect. (**C**) Tension is not uniformly distributed within the structure of the phospholipid bilayer, but is mostly concentrated to two thin layers within the two leaflets. (**D**) The insertion of conical shaped amphipaths into the bilayer may induce a substantial membrane bending effect, change relative thickness of the membrane, or induce local forces acting on the channel protein. Amphipaths may be incorporated into the inner or outer leaflet. (**E**) The pressure gradient between the two sides of the cell-attached or excised membrane patch (*p* = −40 mmHg in the illustration) induces tension, depending on the radius of curvature (r). (**F**) The membrane indentation with a blunt probe (red) imitates the physiological local mechanical effect during a whole-cell patch clamp measurement. (**G**) Cellular swelling in hypotonic environment stretches the plasma membrane. (**H**) The fluid stream causes shear stress of a cell attached to solid support.

From the mechanical point of view, the ion channels are deformable bodies embedded in a predominantly two-dimensional liquid phase of the phospholipid bilayer. The mechanosensitive ion channels are special because mechanical stimulus intensities in the physiological range affect their gating, and the consequent changes in channel activity contribute to the mechanotransduction and physiological response at the cellular or sensory organ level [57]. These proteins normally function in the highly regulated and complex environment of the biological membrane, characterized by the continuously changing interplay of the lipids of the membrane and the transmembrane, cytosolic, and extracellular proteins. Even if we consider a single ion channel in a pure and uniform phospholipid bilayer under experimental conditions, this is a complicated mechanical system, an area of intense investigation, where great advances have been made in the recent years. The mechanical stimuli acting globally on the whole channel protein complex may arise in several varieties (e.g., stretch, bending, shearing, or torsion) (Figure 2A). In addition to these global effects, mechanical stimuli may also target specific molecular components of the channel protein via the localized interaction with other proteins or lipids (Figure 2B). The global effect stretch, also called membrane tension, has generally been examined, whereas the other mechanical actions have been less frequently approached experimentally in the case of the K_2P_ channel proteins.

Similarly to Pascal’s law regarding the pressure in fluids, static membrane tension is generally assumed to be isotropic in the membrane plane in a homogenous system (the tension, measured in mN/m, is the same in all directions, as illustrated by equal red and black *arrows* in Figure 2A) [58]. Nevertheless, the special composition (e.g., lipid raft) may interfere with the free mobility of lipids in the membrane plane, or specific proteins may impose high local membrane curvature and influence tension [59]. As the most simple approximation, according to Laplace’s law in Equation (1), the tension is related to the curvature of the membrane and the pressure difference on the two sides:

(1) $$\begin{array}{c} T=\alpha \times P\times r \end{array}$$

where *T* is the tension; P is the pressure; r is the radius of curvature; and α is a constant determined by the geometry (e.g., 1 for a tube, 0.5 for a sphere). However, in reality, the phospholipid bilayer is not homogenous, and it is plausible that differential tensions may develop in the two leaflets, if the radius of curvature is low in microstructures such as caveloae or microvilli [60,61]. Although it is rarely addressed in the case of K_2P_ mechanosensitive channels [62], dynamic tension may also transiently arise in the membrane, which may be anisotropic in the plane, and results in the rearrangement of the phospholipid molecules, just like the pressure gradient causes flow in 3D fluids. This may be related to the short initial phase of rapid “desensitization” of TREK/TRAAK current after the onset of the mechanical stimulus [62,63].

A growing body of calculations and molecular dynamics simulations suggests that tension is not uniformly distributed within the structures of the phospholipid bilayer, instead, there is intramembrane tension anisotropy [64,65]. Tension is mostly present at the “neck region” of the phospholipid molecules, at the level of the glycerol backbone. The local negative pressure peak of the transmembrane pressure profile in the range of −300 bars (−3 × 10^7^ N/m^2^) would evoke a substantial expanding force on a cylindrical transmembrane protein, as illustrated in Figure 2C. In contrast, in the “tail” and “head” regions of the phospholipids, slight compression of the transmembrane protein occurs. Although this description provides a general insight into the tension distribution in the transmembrane space, K_2P_ channels are not perfect cylindrical structures, and the unique chemical characteristics of the protein surface may influence the transmission of force between the lipids and the channel. Furthermore, the phospholipid bilayer can also be thinned by increased tension, it can be bent by interacting proteins or by the incorporation of asymmetric lipids or other conical shaped amphipaths (Figure 2D). Although the forces acting on the channel in the lateral direction may be the most important, the crenator trinitrophenol and the cup-forming chlorpromazine amphipaths were also reported to substantially influence the activity of TREK channels [29].

### 2.2. Mechanosensitive Properties of K_2P_ Channels as Reflected by the Currently Available Methods

Most methods for the investigation of the mechanosensitive K_2P_ channels modulate membrane tension in general, and in some cases, quantitation of the stretch is possible [66]. However, the above-discussed fine details of the mechanics of channel protein and membrane are not clearly revealed by the data obtained from the usual experimental protocols. Therefore, further methodological development will be required to elucidate which mechanical effects are important at the molecular level, in addition to overall membrane tension, and contribute to channel regulation under physiological conditions.

The pressure-controlled patch clamp method has been widely applied to study the mechanosensitivity of TREK/TRAAK channels. The adjustment of negative (subatmospheric) pressure in the lumen of the patch pipette (Figure 2E), under cell-attached or excised patch conditions, typically increases the K^+^ channel activity. In several reports, the pressure values are given, with the assumption that the membrane patch geometry is similar among the compared groups. Nevertheless, the gating of the mechanosensitive channels is not directly affected by the pressure difference between the two sides of the membrane, but it is determined by the tension of the phospholipid bilayer. Thus, in some experiments, the radius of curvature of the membrane in the patch pipette was also measured, and the actual tension was estimated by using Laplace’s law. These calculations are complicated by the lipid–glass adhesion and capillary tube effects, which result in the significant (non-uniform) tension of the patch membrane even at zero pressure difference [62]. These measurements suggest that TRAAK channels are activated by the membrane tension in the 0.5–12 mN/m range [67]. TREK/TRAAK have low basal activity at the resting tension of the plasma membrane, which may be between 0.01 and 0.3 mN/m [59,68], show remarkable sensitivity to minor tension changes, and the channel activity is gradually increased up to the lytic tension of the plasma membrane. The mechanical threshold for K_2P_ activation is definitely lower than those of the extensively investigated MscS and MscL bacterial mechanosensitive channels, which only open around 5 or 10 mN/m [69,70], respectively, and provide a defense of the prokaryotic cell against hypoosmotic shock. It is more difficult to compare the threshold of TREK/TRAAK with that of the Piezo1 mechanosensitive non-selective cation channel, since both channel types open at low tension levels in the range of the resting membrane tension [71,72,73].

In addition to the threshold, the slope of increasing channel activity in response to more forceful membrane tension is also evidently important as a determinant of mechanotransduction. In this respect, both MscL and Piezo1 appear to be more sensitive than the K_2P_ channels. The half-maximal tension for Piezo1 opening (t_50_ or σ_1/2_) was reported to be 1.4 ± 0.1 mN/m or 4.5  mN/m [71,72,73]. These low values suggest that Piezo1 may activate more steeply in response to the increasing membrane tension than TREK/TRAAK. The low tension threshold of K_2P_ activation ensures sensitivity to physiological mechanostimulation, although the maximal activation at the lytic tension levels also does not exclude the possible protective role of these channels against membrane rupture.

**Figure 3:**
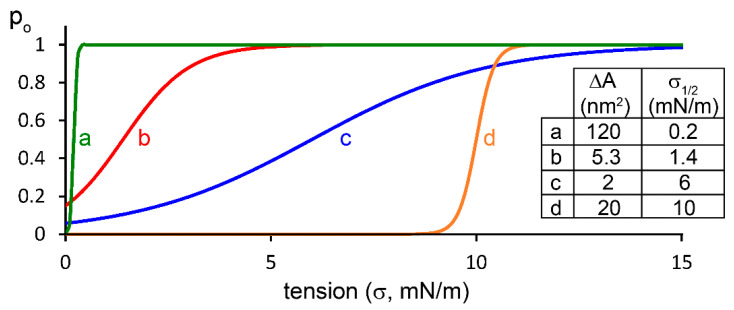
Theoretical mechanosensitivity curves were calculated on the basis of the simple thermodynamic model, considering the change in membrane surface area by channel gating. (a) The theoretical maximum of the change of membrane surface area by Piezo1 gating from structure determinations (ΔA = 120 nm^2^, [76]) was used with an arbitrary value of σ_1/2_ in the resting tension range of the plasma membrane. (b) ΔA and σ_1/2_ values of Piezo1 measured by the pressure controlled patch clamp method were applied [72]. (c) The ΔA estimated for TREK-1 and TRAAK channels by the pressure controlled patch clamp [63] was used with a plausible (arbitrary) value of σ_1/2_. (d) The values accepted for the MscL bacterial mechanosensitive channel were used [69,75].

The simplest thermodynamic theoretical approach to mechanosensitive channels relates the slope of mechanoactivation to the change in membrane surface area occupied by the channel during mechanogating. The channel opened by the tension (open conformation, “O”) occupies a higher membrane surface area than the channel in the resting (closed, “C”) state. The ratio of the number of channels in the closed and open states is determined, in an empirically pleasing way, by the Boltzmann factor as Equation (2):

(2) $$\begin{array}{c} \frac{C}{O}=e^{\frac{-\Delta A\left(\sigma -\sigma_{1/2}\right)}{k_{B}T}} \end{array}$$

where ΔA is the difference of membrane surface area between the closed and open conformations; σ is the actual membrane tension; σ_1/2_ is the membrane tension at which half-maximal channel activation occurs; k_B_ is the Boltzmann constant; and T is the absolute temperature. The consideration is similar to the treatment of the activation of voltage-gated channels, however, here the difference of energy levels of the two conformations is given by the product of tension and change in area, instead of the equivalent gating charge and membrane potential. The change of membrane area (ΔA) by the gating of TREK-1 and TRAAK was estimated to be approximately 2 nm^2^ [63] and 4–5 nm^2^ for TREK-2 [74]. This ΔA was less than those of MscL (20 nm^2^, [75]) or Piezo1 (measured as 5.3 nm^2^, corresponding to Boltzmann ratio 0.8 [72], or suggested to be 120 nm^2^ as a theoretical maximum on the basis of structure determinations [76]), in good accordance with the more moderate slope of mechanosensitivity of TREK/TRAAK channels than those of MscL and Piezo1. Here, the slope of mechanosensitivity means the dependence of the probability of the open state on the tension (Figure 3), practically the slope of the “linear” part of the function as Equation (3):

(3) $$\begin{array}{c} p_{o}=\frac{O}{O+C}=\frac{1}{1+e^{\frac{-\Delta A\left(\sigma -\sigma_{1/2}\right)}{k_{B}T}}} \end{array}$$

The above thermodynamic model accounts for the effect of the changing membrane tension and the lateral forces acting on the channel protein, however, it does not represent a comprehensive theory of mechanosensitivity. Real channels may have several conformational states corresponding to gradually increasing ΔA, and recent studies suggest that the induction of local membrane curvature by the presence of the mechanosensitive channel under resting conditions is a major functional determinant. According to this idea, Piezo1 may be located in a membrane indentation caused by its own deforming effect, and the increased tension flattens this depression, thereby increasing the effective available membrane area, ΔA [76]. Since the diameter of K_2P_ channels changes in the inner leaflet of the membrane during mechanogating (ΔA ≈ 2–5 nm^2^), but the width of the protein shows only small variation at the level of the selectivity filter in the outer leaflet [74], TREK/TRAAK conformational changes inevitably result in membrane bending effects during the gating. The induction of high local membrane curvature may have an impact on the above thermodynamic expression, and may influence the effective ΔA and change σ_1/2_ because of the relative rigidity and elastic property of the protein structure, as suggested for Piezo1. In addition, the local bending of the membrane may introduce another non-negligible tension-dependent energetic component, irrespective of ΔA, which is not accounted for in the above model [76].

Although the induction of local membrane curvature by TREK/TRAAK gating has not been directly demonstrated, there is an ongoing debate about the sensitivity of these channels to the tension difference in the two leaflets of the plasma membrane (sometimes also referred to as the membrane “torque”) [77]. Unexpectedly, it is also controversial whether the TREK/TRAAK channels show asymmetric pressure sensitivity. On one hand, it has been reported that positive and negative pressures identically activate TRAAK and TREK-1, apart from a minor difference, because of the smaller radius of curvature of the same inside-out membrane patch in the case of positive pressures in the pipette [62]. The equal activation of K_2P_ channels by positive and negative pressures is consistent with the almost identical tensions in the two leaflets of the bilayer, irrespective of the direction of the pressure gradient, when the radius of curvature is in the range characteristic for the inside-out membrane patches (i.e., the radius of curvature is relatively large). On the other hand, a truncated version of TREK-2, reconstituted in giant unilaminar vesicles (GUVs) and measured by a planar bilayer patch clamp system, was activated by negative, but not by positive pressures (applied from the ”extracellular” direction). The channels incorporated into the same membrane patch in the opposite orientation also showed asymmetric response, however, with a preference for positive pressures [77]. If the asymmetric response to pressure indeed corresponds to a real biological property of the K_2P_ channels, then these proteins have to be extremely sensitive to the tension difference between the two leaflets. The mechanism could be envisioned as the seesaw-like movement of a transmembrane helix, preferentially influenced by the difference, instead of the absolute value of the tension. Alternatively, the tension difference between the two leaflets can be amplified by the small local radius of membrane curvature around the channel [77].

Whereas the pressure-controlled patch clamp technique supplied the most quantitative data about the global effect of membrane tension on the K_2P_ channels, other methods have also been used to evoke mechanoactivation in a way more reliably approximating certain physiological stimulations. Nevertheless, the close resemblance to the physiological situation comes at a price, and it is almost impossible to relate these results to the direct mechanical changes of the channel protein (e.g., as illustrated in Figure 2A,B). The membrane indentation with a blunt probe during whole cell patch clamp recording (“cell poking” [62,66]) faithfully imitates the direct mechanical stimulation of the cell (Figure 2F). Quantitation of the membrane displacement (and its kinetics) is possible, however, this is not directly proportional to the induced membrane tension, which also varies with cellular geometry and the probe position. The membrane indentation method verified the exquisite mechanosensitivity of the TREK/TRAAK channels independently of the pressure-controlled patch clamp technique, under conditions when the current of the other K_2P_ channel types was completely unaffected by large membrane displacements [62].

The cellular swelling in hypotonic extracellular fluid also resulted in the increase in membrane tension (Figure 2G), and mimics another type of mechanostimulation, which has relevance under physiological conditions. In this method, the membrane tension is slowly changed after solution exchange, and the kinetics of water accumulation in the cell is also affected by several independent factors (e.g., aquaporin content or other ion transport mechanisms of the membrane). The hypotonic challenge causes regulatory compensation and the signaling pathways may also influence the mechanosensitive channel independently of the direct action of the membrane tension. The TREK-1 current was shown to be reduced by hyperosmotic solution in Xenopus oocytes [29], and was suggested to be activated by the extracellular hypoosmotic environment in isolated rat bladder smooth muscle cells [78]. The TRAAK K^+^ current is also activated by the hypotonic challenge [66].

Fluid stream results in laminar shear stress of the cell (Figure 2H), which is especially important in the circulatory system, as exemplified by the endothelium-dependent vasodilation mechanism [79]. TREK-1 is activated by laminar shear stress when expressed in heterologous systems and the cell is exposed to increased flow of perfusion medium [29,80]. The cellular shear stress (Figure 2H) is obviously different from the molecular shearing of the channel protein (Figure 2A), and it may evoke its mechanical effect by the complex combination of local changes of membrane tension and curvature. Although TREK-1 expression has been found in certain (e.g., mesenteric or skin artery) endothelial cells, the flow-induced vasodilation response was intact in the TREK-1 knockout mice [81], suggesting that the mechanical regulation of TREK-1 by the shear stress did not contribute to this vascular effect. Nevertheless, it was suggested that TREK-1 is regulated by the increased aqueous humor shear stress in trabecular meshwork cells in the eye, and this effect may contribute to the regulation of the medically important intraocular pressure value [80].

It has recently been reported that TREK-1 and TRAAK channels are also activated by high intensity ultrasound, as a mechanical effect, and contribute to the hyperpolarization evoked by the application of ultrasound, and to the decreased activity of the targeted neurons in the brain or retina [82,83].

In summary, the members of the TREK subfamily are activated by all the methods designed to stimulate mechanosensitive channels under experimental conditions in isolated cells or membrane patches. There is solid evidence that TREK/TRAAK channels are activated by the increased tension of the plasma membrane via the direct interaction with the channel protein (the “force from lipid” principle is verified).

### 2.3. The Models of TREK/TRAAK Mechanogating

**Figure 4:**
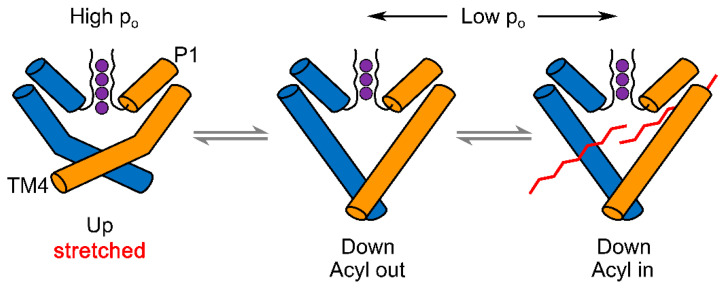
Schematic representation of the conformational changes induced by the mechanogating of K_2P_ channels. The first pore helix (P1) and fourth transmembrane segment (TM4) of the two subunits are shown in orange and blue. The potassium binding sites in the selectivity filter are illustrated as purple spheres. The schematic is based on the crystal structures of TRAAK and TREK-2 [45,67].

The atomic resolution structure determinations [3,5,9,16,45,67,84,85,86] and recent molecular dynamics simulations [74,87,88,89,90,91] provided major insight into the possible mechanogating-induced conformational changes of the TREK/TRAAK channel proteins. A major outcome of these studies is that the mechanogating of K_2P_ channels is related to two pivotal types of conformation, the “up” and “down” states. In the “up” state (as opposed to the “down” state), the cytosolic end of the inner transmembrane helix (TM4) is rotated toward the membrane by about 25 degrees around the flexible glycine “hinge” in the middle of TM4 (Figure 4) [67]. In this way, the intracellular half of TM4 helix becomes more horizontal (more closely parallel to the membrane plane), the diameter and the cross sectional area of the channel in the intracellular leaflet of the lipid bilayer are increased, and TM4 is not straight any more (as in the “down” state), but a kink is formed on its middle part. As a detail of the mechanism, in order to allow this critical structural rearrangement, rotations of TM2, TM3, and TM4 also occur around the respective axes of these helices. The increased membrane tension is associated with the higher probability of the “up” state, since this conformation results in higher membrane surface area, and the increase in ΔA during the conversion from the “down” to the “up” state is energetically advantageous, as detailed in the previous section [67,74,92].

It has also been suggested that the “down” state results in the opening of two side fenestrations of the channel at the level of the hydrophobic core of the phospholipid bilayer [67]. An acyl chain of a phospholipid in the inner leaflet of the membrane may insert into the fenestration and block the pore as another possible mechanism of mechanogating (“the lipid occlusion” model, Figure 4). Although the lever-like “up” and “down” movements of the cytoplasmic end of TM4, and the piston-like “in” and “out” movement of the lipid acyl chain through the fenestrations may influence gating, it is likely that these mechanisms do not directly provide the gate by themselves. TREK/TRAAK channels can also open in the down state (with low probability of the open state), and quaternary ammonium ion blockers can reach their binding site below the selectivity filter when the channels are not activated by the mechanical stimulus [93]. Similarly, rubidium can also reach the selectivity filter from the intracellular side and activate the members of the TREK subfamily in the absence of mechanical stimulation [2,92]. This indicates that the “up” and “down” movements are clearly different from the “helix bundle crossing” gate of K_V_ and K_IR_ tetrameric potassium channels [94,95], and the cytoplasmic half of the transmembrane pore of K_2P_ channels is not constricted sufficiently by the “down” conformation to block ion permeation at this location. Similarly, the lipid acyl chain intruding via the side fenestration may not completely occlude the pore of TREK/TRAAK channels on average, although it may change the hydrophobic character of the central cavity or influence gating at the selectivity filter [74]. The coupling mechanism of the “up” and “down” conformations to the selectivity filter gating is incompletely understood at present.

It is a great achievement of the past decade that the direct regulation of TREK/TRAAK channels by the membrane tension (the “force from lipid” principle) has been firmly established. Nevertheless, the validity of this principle does not exclude the possibility that mechanical stimuli may also influence the TREK/TRAAK channels by other mechanisms. According to the “force from filament” principle, some mechanosensitive ion channels are directly activated by the force transmitted through interacting proteins (e.g., in the case of the TMC1 channel of the inner ear hairs cells and protocadherin 15 of the tip link structure) [96,97,98]. Although such an influential mechanical effect by associated proteins has not been reported for the K_2P_ channels, possible modulation of the mechanosensitivity is suggested by both intra- and extracellular interactions.

It has been reported that the actin cytoskeleton tonically represses TREK-1 mechanosensitivity [99]. However, this does not necessarily indicate direct interaction with the channel, and the effect may be mediated indirectly by the reduction of the membrane tension. The cortical cytoskeleton anchored to the plasma membrane is assumed to relieve the tension of the phospholipid bilayer, just like two parallel connected springs share the force acting on them [59,100]. In fact, the reinforcement of the cytoskeleton may result in the formation of membrane folds with minimal bilayer tension. This may explain why the chemical disruption of the cytoskeleton, or patch excision, increases the mechanosensitivity of K_2P_ channels, compared with the control cell-attached patch conditions. A similar mechanism has been suggested for the regulation of the mechanosensitivity of TREK-1 by polycystin-2 (TRPP2, PKD2 gene) via filamin A and the reinforcement of the cortical actin cytoskeleton lattice [61,101]. The modulation of the local curvature of the membrane folds by the regulation of the cytoskeleton may have a substantial effect on the membrane tension, according to Laplace’s law. The multimeric form of the extracellular protein cochlin was also reported to inhibit TREK-1 current, and contribute to the elongation of trabecular meshwork cells and impairment of aqueous humor outflow in the eye [80].

In addition to membrane tension and extra- or intracellular tethers, a third possible way of mechanoactivation is the indirect regulation by signaling mechanisms (“force through signaling”). According to this idea, the channel is not the mechanosensor itself, but is regulated indirectly because of the activation of another sensor. This mechanism is not likely to be exclusively responsible for the mechanosensitivity of TREK/TRAAK channels, since the purified K_2P_ proteins also constitute functional mechanosensitive channels in artificial phospholipid membranes, where other mechanosensors and signaling components are not present [62,77]. However, the “force through signaling” hypothesis appears to be attractive as an auxiliary mechanism regarding the polymodal regulation of TREK/TRAAK channels. Phospholipase D2 (PLD2) interacts with the C-terminus of TREK-1 and TREK-2 (but not TRAAK) and locally produces phosphatidic acid (PA), which is an activator of the TREK channels [102]. The redistribution of PLD2 between lipid nanodomains in response to the mechanical stimulus has been suggested to contribute to the mechanosensitivity of TREK channels [103,104].

The mechanosensitivity of TREK-1, TREK-2, and TRAAK homodimer channels has been extensively investigated, however, the TREK subfamily of K_2P_ channels also includes three further channel types, constituted by the heterodimerization of the subunits [105,106,107]. The in vivo contribution of TREK-1/TREK-2, TREK-1/TRAAK, and TREK-2/TRAAK to the background K^+^ current, and the mechanosensitivity of these heteromeric channels remain to be examined. 

In summary, TREK/TRAAK channels are directly regulated by the tension of the surrounding phospholipid bilayer. The modulating effects of extra- and intracellular proteins, and other signaling pathways may complement this principal mechanism. Further studies are required for a better understanding of how the mechanical stimuli result in the conformation changes of the mechanosensor regions of the channels, and how these regions are coupled to the gating at the selectivity filter.

## 3. The Physiological Roles of K_2P_ Channel Mechanical Activation

**Figure 5:**
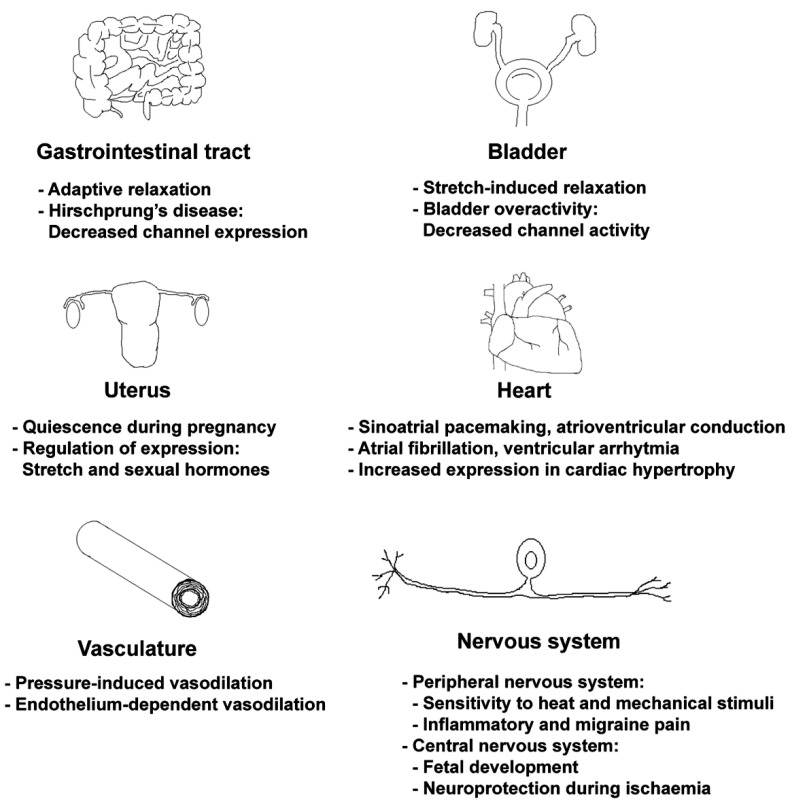
A summary of the physiological and pathophysiological roles of mechanosensitive K_2P_ channels in different organs. For further details, see the corresponding text.

In this section, we primarily focus on the TREK/TRAAK functions related to mechano-activation and consider some interesting examples (summarized in Figure 5), but do not attempt to overview the extensive literature about the role of these channels in general (patho)physiology. While the mechanically induced hyperpolarization via TREK/TRAAK activation plausibly contributes to the physiological processes in some cell types, in other cases, the results are controversial, or the explanation of the observations may spread to the field of exciting hypotheses. One conspicuous problem, difficult to reconcile with the initial expectations, is the co-expression of mechanosensitive K_2P_ and non-selective cation channels in several locations, where the mechanical stimuli evoke depolarization. The mechanical activation of K_2P_ channels hinders the depolarization in these cases, and the biological rationale for this apparently counterproductive effect is not evident. Although it is tempting to speculate, we cannot propose the exact solution of this conundrum at present, since no unified theory based on solid experimental data has been reported about the question to our knowledge.

### 3.1. Hyperpolarization and Relaxation of Smooth Muscle Cells in the Wall of Hollow Visceral Organs

In several hollow organs, a characteristic response to passive distension and stretch of the wall is the relaxation of smooth muscle, which assists further dilation and prevents the unnecessary increase of pressure in the lumen. This response supports the storage function of the hollow organ, in a regulated manner, under certain conditions, and for a given time period. The mechanosensitive K_2P_ channels perfectly fit into this simple reaction. The mechanical stimulus causes the increased tension of the smooth muscle cell plasma membrane, resulting in K_2P_ channel opening and hyperpolarization, followed by the decreased activity of voltage-gated L-type calcium channels, reduced cytoplasmic [Ca^2+^], and muscle relaxation. While this sequence of events provides a general framework, the contribution of K_2P_ channels to the regulation of contractility in these organs may be more complex.

#### 3.1.1. Adaptive Relaxation in the Gastrointestinal Tract

The presence of mRNA coding for TREK family subunits in the gastrointestinal system has been reported in multiple studies [108,109,110,111,112,113]. Immunofluorescence experiments showed cell-type specific expression of the different TREK subunits: TREK-1 is expressed in the smooth muscle and epithelial cells of mouse ileum and colon, while TREK-2 and TRAAK are found in the myenteric plexus neurons of the enteric nervous system. In organ bath experiments, TREK-1/2 channel activators (BL-1249 or riluzole) caused the relaxation of mouse ileum segments that had been precontracted with KCl or carbachol [111]. TREK channel activation reduced the amplitude of spontaneous contractions without influencing the frequency. The relaxation was not influenced by the pretreatment of the organ with tetrodotoxin, suggesting that the effect was mediated by the hyperpolarization of the smooth muscle cells and not by the neurons of the enteric nervous system [111]. Direct activation of endogenous TREK-1 channels by the elongation of murine colonic smooth muscle cells with two attached microelectrodes (mimicking colon extension) was also demonstrated [114], suggesting that TREK-1 may contribute to the mechanically induced hyperpolarization in vivo. Decreased expression of TREK-1 and TRAAK have been reported in Hirschprung’s disease, a condition characterized by the disorder of intestinal motility [112,113].

#### 3.1.2. Distension of the Urinary Bladder

TREK-1 expression was described at both the mRNA and protein level in the smooth muscle cells of the urinary bladder [78,115,116,117,118,119]. Functional importance of the channel was confirmed using electrophysiology on smooth muscle cells and myography performed on isolated strips of muscle [115,116,117]. Stretch applied to detrusor strips of TREK-1 knockout mice increased the amplitude of spontaneous contractions more than in the wild type animals [118]. TREK-1 activity in the detrusor muscle also proved to be a major determinant of relaxation in the human bladder [115]. Decreased levels of channel protein and arachidonic acid-activated K^+^ currents were observed in the muscle strips of patients suffering from detrusor overactivity [116]. In a recent genetic study of patients with symptoms of overactive lower urinary tract, a single nucleotide polymorphism (SNP) leading to a missense mutation (S147F) near the pore domain of TREK-1 was identified, however, the effect of this mutation on the channel activity has not yet been investigated [120]. In good accordance with the human data, chronic (14 day) partial outlet obstruction in wild-type mice results in significantly decreased expression of TREK-1 in the detrusor muscle, and bladder overactivity [119]. Altogether, the experimental data suggest that TREK-1 contributes to the stretch-induced urinary bladder relaxation in the filling phase and may underlie myogenic bladder dysfunction in humans.

#### 3.1.3. Relaxation of the Myometrium during Pregnancy

It has been reported that TREK-1 and TRAAK mRNA and protein are expressed in the rodent and human myometrium [121,122,123,124]. TREK/TRAAK-like functional activity was detected by patch clamp electrophysiology [121,122,123] and myography [121,123,125]. The expression of the channel was found to be increased in pregnant myometrium compared to non-pregnant uterus [121,123,124]. Increased TREK-1 mRNA expression was also observed in isolated muscle strips after prolonged stretch in vitro [124]. This increased expression was not seen in samples obtained after parturition, which suggests that the enhanced TREK/TRAAK activity may contribute to the maintenance of uterine quiescence during gestation [121,123]. Treatment of isolated rat myometrium with progesterone increased the expression of TREK-1 [125], while ovarectomy of mice led to the decreased abundance of the channel [123], raising the possibility that the changes in sexual hormone production during pregnancy play a role in the regulation of the availability of stretch-sensitive K_2P_ channel subunits.

### 3.2. Putative Role of the Mechanical Activation of K_2P_ Channels in the Cardiovascular System

Certain elements in the cardiovascular system (e.g., arteries, arterioles, and cardiac left ventricle) are characterized by high and/or rapidly changing pressure values (normally up to 120 mmHg), which can evidently produce enough tension of the vessel or ventricular wall to activate mechanosensitive ion channels. Nevertheless, even in other regions (e.g., capillaries, venules and veins of the systemic circulation, cardiac atria, or the elements of the pulmonary circulation), the relatively low pressures, which may be in the 2–35 mmHg range, may also be sufficient to cause physiologically relevant tension and activation of mechanosensitive channels. It is well established in physiology textbooks that the transmural pressure and tension of the vessel wall, or the flow and resulting shear stress evoke robust and regionally diverse responses in the cardiovascular system. However, the mechanosensitive ion channels involved in these critically important circulatory responses are not generally known, and have only just begun to be explored at the molecular level. As an elegant example, it has recently been reported that the baroreceptor mechanosensitivity, and thus the short-term regulation of arterial blood pressure, are based on the local activation of Piezo1 and Piezo2 channels in the sensory nerve endings in the aortic arch and carotid sinus [126].

The identification of mechanosensitive channels in the cardiovascular system remains a substantial methodological challenge, which may require organ- or even cell type-specific knockout models to discriminate between the roles of the channels in the different tissues [126,127]. K_2P_ channels are expressed in multiple structures in the cardiovascular system (see below), making it difficult to distinguish their functions in different cell types. In fact, the function of mechanosensitive K_2P_ channels does not fit well theoretically into the classic vascular reactions at all. For example, in the Bayliss effect, which contributes to the autoregulation of arteriole diameter and resistance via the contraction of vascular smooth muscle in response to increased luminal pressure, the mechanical activation of K_2P_ channels would hinder the principal effect by causing hyperpolarization and relaxation. Similarly, in the endothelium-dependent flow-induced vasodilation mechanism, the activation of TREK channels by the shear stress of the endothelial cells would counter the calcium signal and nitric-oxide production, again, in contrast to the expected result. Accordingly, it has been reported that the myogenic contraction response to stepwise increases in intraluminal pressure and flow-induced dilation of mesenteric arteries were not affected in the TREK-1 knockout mice [81]. Therefore, it remains an open question whether the TREK channels contribute to the regulation of diameter by mechanical stimuli in the vasculature. The role of TREK mechanosensitivity has not yet been directly examined under conditions when the stretch causes dilation such as in the case of the breakdown phase of the Bayliss effect by pathologically high pressures, or the pressure-induced vasodilation of pulmonary arteries.

#### 3.2.1. Mechanical Activation of K_2P_ Channels in the Heart

The general (patho)physiology of cardiac mechanosensitive K_2P_ channels has recently been reviewed in a comprehensive manner [128,129,130]. It has been reported that TREK-1 (but not TREK-2 or TRAAK) is expressed in the heart of several different species at the mRNA and protein level (for summary, see [128]). TREK-1 is present in both atrial and ventricular cardiomyocytes, and also in the nodal tissues [128]. TREK-1 is differentially expressed between the endo- and epicardial regions of the left ventricular wall, 17-fold higher expression of TREK-1 was reported in the endocardial, compared to the epicardial muscle in rats [131,132,133,134]. It was recognized early after the discovery of the channel that several properties of TREK-1 very well corresponded to the stretch-activated potassium current [135], which has long been described in atrial and ventricular myocytes [136,137]. Afterward, the contribution of TREK-1 to the mechanoelectrical feedback mechanism in the heart has been hypothesized [132]. According to this hypothesis, the strain of the myocardium activates TREK-1 channels, and the consequent hyperpolarization of the cardiomyocytes modulates the electrical events (e.g., it may shorten the action potential duration, slow the propagation of excitation, and even prove to be arrhythmogenic under pathological conditions) [138]. Despite the early and logical formulation of the hypothesis, direct experimental evidence in support of the contribution of TREK-1 to the mechanoelectrical feedback has not yet been produced. The investigation of TREK-1 is complicated by the parallel activation of a Ca^2+^-permeable non-specific cation channel in response to moderate stretch of the myocardium (also called SAC, stretch activated channel), however, SAC has not been unequivocally identified at the molecular level (for review, see [138,139]). Another important physiological regulatory mechanism is the control of ANP (atrial natriuretic peptide) release by the stretch of the atrial wall, although the possible role of TREK-1 in this endocrine effect has not been reported. It has recently been suggested that TREK-1 contributes to the mechanosensitive response of human heart valve interstitial cells, by which the integrity of valve cusps is chronically maintained [140].

TREK-1 channels play an important role in regulating both sinoatrial pacemaking and atrioventricular conduction. The direct role of TREK-1 in sinoatrial pacemaking was demonstrated using a cardiomyocyte-specific TREK-1 knockout mouse model [141]. Deletion of TREK-1 leads to a decrease in background potassium currents, a depolarized maximum diastolic potential, and impaired repolarization in the cells of the sinoatrial node, resulting in increased rate of action potential firing. Interestingly, these electrophysiological changes did not result in increased intrinsic heart rate of the TREK-1 knockout animals, which could be explained by the compensatory alteration of autonomic neuron activity. However, these animals suffered from exercise-induced sick sinus syndrome. A similar phenotype was observed when trafficking of the channel to the plasma membrane was impaired by deletion of the TREK-1 interacting partners βIV spectrin or the Popeye-domain containing proteins POPDC1 and POPDC2 [142,143]. Furthermore, POPDC1 and POPDC2 double knockout animals exhibited signs of atrioventricular conduction disorder (AV block). The mutations disrupting the regulation of TREK-1 by POPDC1 or POPDC2 proteins lead to the development of familial AV block [144,145]. Thus, the important role of TREK-1 in the function of the sinoatrial and/or atrioventricular nodes is established, however, the contribution of this channel to the local stretch-induced changes of the heart rate and atrioventricular conduction velocity is not known. There is a substantial interspecies difference; the heart rate is increased by the local stretch of the sinoatrial node in rabbits and most probably in humans, but it is not reproducibly changed in mice [138,146]. TREK-1 activation would counter this effect in the species, where the heart rate is increased by the local stretch of the sinoatrial node.

The potential role of TREK-1 in the generation of cardiac arrhythmias has been investigated using both animal models and human tissue samples obtained from patients with heart disease. A decrease in TREK-1 expression has been shown in both a murine transgenic model known to develop spontaneous atrial fibrillation [147], and porcine models in which atrial fibrillation was induced by rapid electrical pacing via an implanted pacemaker [148,149]. In the porcine model, adenovirus-mediated expression of TREK-1 reversed the observed phenotype. In humans, the degree of TREK-1 downregulation differed between studies [147,150], which can potentially be explained by differing clinical parameters (e.g., presence or absence of heart failure) of the examined cohorts. However, a trend toward reduced TREK-1 expression was always observed. TREK-1 may also play a role in the generation of ventricular arrhythmias (for a detailed review, see [139]).

Right ventricular outflow tract tachycardia was recently reported to develop as a consequence of a gain of function mutation of TREK-1, discovered by whole-exome sequencing of a patient [151]. The mutation (I267T) is close to the selectivity filter of the channel and results in a remarkable change in the ion selectivity; the K^+^-selective pore of TREK-1 becomes permeable to Na^+^ ions. Furthermore, the mutation increased the sensitivity of the channel to membrane stretch. The β-adrenergic regulation of I267T mutant TREK-1 is also influenced: while wild-type TREK-1 is inhibited, the Na^+^ current of the I267T mutant TREK-1 is augmented by β1-receptor stimulation.

Upregulation of TREK-1 mRNA expression and a subsequent increase in TREK-1 like currents in ventricular cardiomyocytes was observed under conditions of cardiac hypertrophy in both rats and mice [134,152]. Furthermore, cardiac hypertrophy leads to a significant increase in the transmural expression gradient of TREK-1 (i.e., higher endocardial expression compared to the epicardial region). In a recent study, it has been shown that the global deficiency of TREK-1 in mice increases the hypertrophy observed after pressure overload, but no signs of cardiac dysfunction were apparent in the absence of pressure overload in the TREK-1 knockout animals [127]. Intriguingly, cardiomyocyte-specific deletion of TREK-1 did not protect against cardiac dysfunction, while the deletion of TREK-1 in fibroblasts led to the preservation of cardiac function after pressure overload. The loss of TREK-1 decreased the fibroblast function and significantly reduced the degree of cardiac fibrosis after pressure overload. These results suggest that the inhibition of TREK-1 activity in fibroblasts could have cardioprotective effects against cardiac dysfunction induced by pressure overload. In contrast, it has recently been suggested that TREK-1 evokes cardioprotective effects in a mice model of ischemia-reperfusion-induced injury [153].

#### 3.2.2. Mechanical Activation of K_2P_ Channels in the Vasculature

The experimental data on the significance of the mechanical activation of TREK/TRAAK channels in the vasculature are limited. We mention some results, which may be related to the channel activation by membrane tension or bending.

The expression of TREK-1 was reported in the different regions of the systemic and pulmonary circulation in both endothelial and smooth muscle cells. TREK-1 is present in the mesenteric arteries of rats and mice [154]. As mentioned above, the channel does not contribute to the myogenic contraction induced by increased intraluminal pressure, however, the acetyl-choline- and bradikinin-dependent vasodilation is impaired in TREK-1 knockout animals [81]. In addition to mesenteric arteries, TREK-1 is also expressed in the microvessels of the cutaneous circulation, and immunofluorescence experiments have shown the presence of TREK-1 in vascular smooth muscle cells and endothelial cells. Cutaneous pressure-induced, or acetyl-choline- and bradykinin-dependent vasodilation was compromised in TREK-1 knockout animals, suggesting that TREK-1 is required for the endothelial function in this vascular bed [81].

It has initially been reported that the application of polyunsaturated fatty acids (PUFA) activates the TREK-1 channel not only in neurons, but also in vascular smooth muscle cells of the cerebrovascular circulation in mice and rats [155], and leads to vasodilation and increased cerebral blood flow [155,156]. Both PUFA-dependent and endothelial receptor-mediated vasodilation was impaired in TREK-1 knockout animals compared to wild-type controls [156]. Nevertheless, the functional importance of TREK-1 in the PUFA-dependent vasodilation in the cerebrovascular circulation remains controversial, because the altered dilation of the basilar artery by alpha-linolenic acid could not be detected in another TREK-1 knockout mouse strain by another research group [157].

Most vessels in the systemic circulation respond to hypoxia by vasodilation. In contrast, in the placental and pulmonary circulation, vessels constrict under hypoxic conditions. Interestingly, TREK-1 expression has also been shown in both the placental circulation (immunofluorescence experiments) and pulmonary arteries (mRNA expression in mice and rats) [108,158,159,160]. To date, the functional role of TREK-1 in pulmonary circulation has not been examined. In wire myography experiments, the application of the non-specific TREK-1 activator riluzole led to a dose-dependent relaxation of placental arteries [161].

### 3.3. Mechanical Activation of K_2P_ Channels in the Nervous System

The members of the TREK subfamily of K_2P_ channels are expressed in a wide variety of neurons in both the peripheral and central nervous system. In the peripheral system, in the mechanosensitive primary sensory neurons, the response to mechanical stimuli is influenced by the genetic deletion of TREK/TRAAK channels (see the details below). However, it is uncertain whether the mechanosensitivity of the K_2P_ channels is directly involved in the detection of mechanical stimuli, or the changes are simply consequences of the elimination (or the altered composition) of the background K^+^ conductance in the genetically modified animals. In the central nervous system, the mechanosensitive channels may be important during neurodevelopment, or under pathological conditions (e.g., in the case of neuronal swelling in ischemia). Nevertheless, there is solid evidence that the currents of the members of the TREK subfamily substantially contribute to the neuronal function under normal condition, after the development of the central nervous system, raising the suspicion that mechanical gating is not always the primary regulator of these K_2P_ channels.

#### 3.3.1. Peripheral Nervous System

The expression of all TREK/TRAAK subunits has been shown at both the mRNA and protein level in the primary sensory neurons of the dorsal root and trigeminal ganglia [162,163]. In situ hybridization and immunofluorescence experiments indicated the high expression of TREK/TRAAK channels in small- and medium-diameter nociceptive sensory neurons [164]. The sensitivity of the TREK subfamily to both temperature and force suggests that these K_2P_ channels might play a role in the detection of heat and mechanical stimuli. Furthermore, the co-expression of TREK/TRAAK subunits and other ion channels involved in thermosensation and nociception (e.g., the capsaicin receptor, TRPV1) has been confirmed using single-cell RNA sequencing [165,166].

The contribution of TREK family subunits to the regulation of sensory neuronal activity has mainly been analyzed using knockout animal models, in the absence of sufficiently specific pharmacological tools. Deletion of TREK-1 or TREK-2 led to an increased sensitivity to acute low-intensity (warm) thermal stimuli, while responses to noxious heat were similar in the wild type, TREK-1, and TREK-2 knockout animals [164,167]. Deletion of TREK-2 led to increased behavioral responses to warm temperatures, however, the perception of noxious cold was unchanged. Combined loss of TREK-1 and TRAAK led to augmented cold-sensitive behavior in noxious cold [168]. These results highlight the distinct, but partially overlapping role of TREK/TRAAK channels in the detection of the environmental temperature.

Deletion of either TREK subunit led to an increase in the sensitivity to low threshold mechanical stimuli, as determined by using von Frey filaments [164,167,168]. The effect can be explained by the unopposed activation of mechanosensitive nonselective cation channels in the absence of the hyperpolarizing influence by TREK. The deletion of TREK-1 or TREK-2 substantially decreased the sensitivity of the animals to another type of stimulus; the painful sensation in response to the injection of hyperosmotic solutions was reduced. The underlying mechanism of this phenotype is not clearly understood at present. This phenotype was also observed after the sensitization of the animals with injections of the inflammatory mediator prostaglandin E2 (PGE2), which intensified the behavioral response to the injection of mildly hypertonic NaCl solution in wild type animals, but much less in the TREK/TRAAK knockouts [164,167,168]. The osmotic pain sensitivity of TRAAK knockout animals was not affected, however, the deletion of TREK-2, or the combined deletion of TREK-1/TRAAK, diminished the response to the injection of hyperosmotic solution after PGE2 sensitization [167,168]. In an animal model of migraine, the injection with the NO-donor isosorbide dinitrate (ISDN), a known migraine trigger, resulted in the increased sensitivity to mechanical stimuli (mechanical allodynia, a common symptom of migraine headache) in animals deficient in both TREK-1 and TREK-2, compared to their wild-type littermates [169]. In rats, knockdown of TREK-2 increased the nocifensive behaviors observed in a different model of inflammatory pain (injection of complete Freund’s adjuvant) [170]. Furthermore, the pharmacological activation of TREK channels decreased the dorsal root ganglion (DRG) or trigeminal ganglion (TG) neuron excitability in current clamp [171], and calcium imaging experiments [172] (and unpublished results from our laboratory). Therefore, the pharmacological targeting of TREK channels could be a new approach to the treatment of migraine and potentially other modalities of pain.

#### 3.3.2. Central Nervous System

All three members of the TREK subfamily are expressed in the brain at all stages of development [50,173,174]. During the fetal and early postnatal development of the nervous system, dynamic reorganization of the cytoskeleton and changes in cell shape are needed for the proper axonal pathfinding, dendritic arborization, and formation of neural networks. The regulation of TREK channels by both membrane stretch and elements of the cytoskeleton gave rise to the hypothesis that TREK channels might be involved in the regulation of neuronal morphogenesis. Increased expression of TREK-1 (or TREK-2) in cultured neurons increased the number of neuronal growth cones, while neurons isolated from TREK-1 knockout mice had a decreased number of growth cones compared to wild-type controls [99]. Surprisingly, no gross abnormalities of the brain were observed in TREK-1 knockout animals, suggesting that the function of TREK-1 may have been substituted by other ion channels during development [175]. Human genetic data are also available that suggest the potential importance of the TREK subfamily in the proper development of the nervous system. Missense mutations in the gene encoding TRAAK (A172E, A244P) were identified in multiple patients suffering from a complex neurodevelopmental disorder [176]. When expressed in a heterologous expression system, these mutant channels had increased basal currents and impaired activation by pressure and arachidonic acid compared to the wild-type channel, suggesting that regulation of TRAAK activity by mechanical forces and lipid mediators might be of importance during nervous system development.

Increased cell volume is one of the earliest pathological consequences of cerebral ischemia and is known to activate TREK family channels. Activation of a background potassium current (and the consequent hyperpolarization) can decrease both the frequency of action potential firing and synaptic activity, which may be beneficial under ischemic conditions, and in other pathologic situations when neuronal activity is increased (e.g., seizures). Furthermore, polyunsaturated fatty acids (which are well-known for their neuroprotective effects) are characteristic activators of TREK channels [19,29,30]. Altogether, these properties of the subfamily urged detailed investigation of their potential role in neuroprotection using TREK-1 and TRAAK knockout animals. TREK-1 knockout mice showed more severe symptoms and an increased death rate in animal models of cerebral and spinal cord ischemia compared to the wild-type controls [175]. TREK-1 is necessary for the neuroprotective effects of PUFAs; deletion of TRAAK did not influence the phenotype observed, which is in good agreement with the lower and less widespread expression of the channel compared to TREK-1. The importance of TREK-1 in the control of epileptogenesis was examined in mice by evoking seizures via intraperitoneal injection of a glutamatergic agonist (kainic acid) or a gabaergic antagonist (pentylenetetrazol, PTZ). In animals lacking TREK-1, seizure activity was more pronounced (based on a subjective clinical scoring system and analysis of EEG recordings) with a corresponding lower rate of survival compared to the control animals [175]. In rats, TREK-1 and TREK-2 were shown to be involved in the neuroprotective effects of isoflurane preconditioning [41,42,177,178,179]. Repeated inhalation of sevoflurane led to an increase in TREK channel functional expression and improved neurological outcomes after evoking cerebral ischemia, however, silencing of the TREK channels greatly decreased the neuroprotective effects of sevoflurane preconditioning [42,180,181].

### 3.4. Physiological Role of the Mechanical Activation of K_2P_ Channels in Other Locations

Pressure gradients exceeding 10 mmHg may arise in the respiratory system under physiological, and especially, under pathologic conditions. In principle, the resulting tensions of the cell membranes may activate mechanosensitive channels. TREK-1 was reported to be expressed in alveolar epithelial cells [182,183], contribute to the reaction to hyperoxia [184,185], and modulate inflammatory mediator (interleukin-6, monocyte chemotactic protein-1) release [186,187]. It has also been suggested that TREK-1 modulates stretch-induced detachment of alveolar epithelial cells [188]. It remains to be established whether mechanically induced TREK-1 activation influences the ion and fluid secretion, cytoskeletal rearrangements, and cytokine production of alveolar epithelial cells.

Normal intraocular pressure (IOP) in the range of 10–20 mmHg drives the reabsorption of aqueous humor through the trabecular meshwork and canal of Schlemm into the venous circulation. TREK-1 may have a function in the regulation of the medically important IOP, which is increased in glaucoma. TREK-1 is expressed in the trabecular meshwork cells [80,189]. It has recently been suggested that the mechanical effect of the fluid flow through the narrow paths activates TREK-1, and TREK-1 (and TRPV4 cation) channels are responsible for the pressure sensitivity and control of calcium signaling in trabecular meshwork cells [190,191].

## 4. Conclusions

It has been established that TREK-1, TREK-2, and TRAAK are mechanosensitive background K^+^ channels directly regulated by the tension of the plasma membrane. This direct mechanism may be complemented by the forces exerted via interacting proteins and the modulation of the membrane tension by the cytoskeleton. The intracellular signaling pathways (e.g., resulting in channel phosphorylation or the altered composition of the phospholipid environment) have critical importance in the regulation of mechanosensitivity, and perhaps also contribute to the propagation of signals from other mechano-dependent sensors to TREK/TRAAK channels.

The fundamental conformational changes of the channel protein in response to membrane stretch have been identified by atomic resolution crystal structure determinations and analyzed by molecular dynamics simulations. The bending of the fourth transmembrane segment results in a conformational state with higher surface area of the channel in the membrane plane, which is energetically favored when the membrane tension is increased. Most probably, the mechanosensor elements of the channel structure (e.g., TM4), which are directly rearranged by the membrane stretch, do not constitute the gate by themselves, but rather the effect is transmitted to the selectivity filter, the primary site and general mediator of K_2P_ gating. The mechanism of gating at the selectivity filter, and the coupling of the activation of mechanosensor regions to the K_2P_ channel opening remain to be further examined.

A growing body of evidence indicates the importance of TREK/TRAAK channels in several physiological and pathological processes. However, only a small fraction of these studies support the role of the mechanical activation of the channels directly. It remains a substantial methodological challenge to establish the causal relationships between the mechanical stimulus, TREK/TRAAK activation, and the alteration of the examined function. As the understanding of the molecular mechanisms of TREK/TRAAK mechanosensitivity is improved with the development of more specific pharmacological tools and targeted genetically modified animal models, the contribution of the mechanoactivation to the function of the cardiovascular and nervous system will be elucidated.

## Data Availability

Data sharing not applicable. No new data were created or analyzed in this review.
